# Ultrasound‐guided interstitial photothermal therapy generates improved treatment responses in a 9464D model of neuroblastoma

**DOI:** 10.1002/btm2.10749

**Published:** 2025-01-06

**Authors:** Grace E. Olsson, Rohan V. Patil, Samantha J. Chin, Katharine N. Rus, Elizabeth E. Sweeney, Karun V. Sharma, Rohan Fernandes

**Affiliations:** ^1^ George Washington Cancer Center, School of Medicine and Health Sciences George Washington University Washington, DC USA; ^2^ School of Medicine and Health Sciences George Washington University Washington, DC USA; ^3^ The Integrated Biomedical Sciences Program, School of Medicine and Health Sciences George Washington University Washington, DC USA; ^4^ Department of Biochemistry & Molecular Medicine, School of Medicine and Health Sciences George Washington University Washington, DC USA; ^5^ Department of Interventional Radiology Children's National Hospital Washington, DC USA; ^6^ Department of Medicine, School of Medicine and Health Sciences George Washington University Washington, DC USA

**Keywords:** interstitial laser, neuroblastoma, photothermal therapy, Prussian blue nanoparticles, ultrasound imaging

## Abstract

We describe the use of ultrasound image guidance to improve treatment outcomes when administering interstitial photothermal therapy (I‐PTT), an experimental cancer treatment modality. I‐PTT is a promising thermal therapy for tumors using intratumorally injected nanoparticle‐based photothermal agents activated by an interstitially placed laser diffuser. We hypothesized that ultrasound‐based image guidance yields improved tumor treatment outcomes in terms of tumor regression and survival by improving the accuracy of the placement of the laser fiber and nanoparticles within a tumor and facilitating more precise PTT delivery. To test this hypothesis, we assessed the effect of ultrasound‐guided I‐PTT (US I‐PTT) on neuroblastoma, an aggressive solid tumor of childhood, using the 9464D syngeneic model in C57BL/6 mice. US I‐PTT using Prussian blue nanoparticles activated by an interstitial cylindrical laser diffuser generated an equivalent in vivo thermal dose as blinded, non‐image‐guided I‐PTT (B I‐PTT). However, US I‐PTT resulted in significantly higher treatment accuracy compared to B I‐PTT, attributable to the image guidance. Importantly, this improved accuracy translated to improved treatment outcomes wherein mice treated with US I‐PTT exhibited significantly improved tumor regression, tumor‐free survival, and long‐term survival compared to mice treated with B I‐PTT. Further, histological analyses of the tumors post‐PTT confirmed the advantages conferred by US I‐PTT over B I‐PTT for tumor control. These proof‐of‐concept results demonstrate the value of using ultrasound guidance for I‐PTT treatment and the translational implications of this approach to provide a more accurate and effective treatment for neuroblastoma.

**Translational Impact Statement**

Nanoparticle‐based photothermal therapy (PTT) shows impressive treatment results in preclinical animal models of cancer, but its clinical adoption is limited. This study uses ultrasound imaging guidance to improve PTT and may improve its clinical translation. The benefits of using ultrasound guidance to improve interstitial PTT treatment outcomes for neuroblastoma, as demonstrated here, could facilitate follow‐up studies using ultrasound as an integral component of image‐guided PTT on its path to more widespread clinical adoption.

## 1 INTRODUCTION

Photothermal therapy (PTT) is a nanoparticle‐mediated thermal therapy for treating cancer.^1^, ^2^, ^3^ Preclinically, Prussian blue nanoparticles (PBNPs) have been extensively utilized by others^1^, ^4^, ^5^, ^6^, ^7^, ^8^, ^9^ and us^2^, ^3^, ^10^, ^11^, ^12^, ^13^, ^14^, ^15^, ^16^, ^17^, ^18^, ^19^, ^20^ as agents of PTT because they absorb light in the near infrared (NIR) wavelength range^21^ and generate heat when illuminated by light at these wavelengths.^2^, ^19^ In addition, PBNPs are easily synthesized on a large scale at low costs and have been granted U.S. Food and Drug Administration approval in decorporation applications.^22^, ^23^ PBNP‐PTT has also been used in the literature as a simultaneous diagnostic tool wherein PBNPs are used for their imaging capacity (both alone and in combination with imaging agents).^4^, ^5^, ^6^, ^7^, ^8^, ^9^ We have demonstrated the use of PBNP‐PTT in combination with chemotherapeutic agents^10^ and antibody‐,^11^, ^14^, ^15^ agonist‐,^16^, ^18^ and cell‐based^24^, ^25^ immunotherapies, suggesting its multi‐functional use as a tool for cancer therapy. However, most of the existing studies in the literature involving PBNP‐PTT describe superficial PTT (S‐PTT),^2^, ^10^, ^15^, ^26^ where an external beam laser is illuminated onto the surface of a tumor containing injected or accumulated PBNPs. S‐PTT has shown efficacy in ablating tumors, but this strategy comes with intrinsic limitations, particularly in its inability to reach deeper tumors and the non‐uniform heating it confers on a treated tumor.^3^ Interstitial PTT (I‐PTT) is an alternative approach that utilizes a laser fiber placed intratumorally (i.t.) to activate the nanoparticles instead of an external beam laser superficially.^27^, ^28^ This enables the tumor to be photothermally heated from the “inside out.”^27^, ^29^ In a previous study, we demonstrated that I‐PTT using PBNPs heats more uniformly than S‐PTT in murine models of neuroblastoma.^3^ Additionally, mice treated with I‐PTT survived significantly longer than mice treated with S‐PTT or controls. These proof‐of‐concept findings highlight the superior efficacy of I‐PTT compared to S‐PTT in the treatment of neuroblastoma,^3^ while also suggesting its potential relevance to a wider spectrum of solid tumors.

Despite these promising findings, I‐PTT has limitations that serve as barriers to clinical translation. Even if used for superficial or accessible tumors, there is the potential for I‐PTT to miss treatment zones, which could drive tumor recurrence^30^, ^31^ and treatment failure because it is administered without the benefit of image guidance. Therefore, utilizing imaging modalities to administer I‐PTT to tumors may be required to yield more effective I‐PTT treatment outcomes. In this context, ultrasound imaging represents a promising modality for guiding I‐PTT since it is a widely utilized, cost‐effective clinical imaging modality. Here, we test if ultrasound‐guided I‐PTT (US I‐PTT) improves treatment outcomes for neuroblastoma, a common pediatric cancer that accounts for 15% of childhood cancer‐related deaths. Patients with high‐risk neuroblastoma have particularly poor prognoses, with tumor recurrence in 50% of cases after intensive, standards‐of‐care treatment regimens.^32^, ^33^, ^34^, ^35^ Neuroblastoma typically manifests in and around the adrenal glands, kidneys, and other deep‐seated regions of the abdomen. Further, US imaging is already commonly used in the diagnosis and monitoring of neuroblastoma and is relatively facile and cost‐effective in comparison to other common imaging modalities.^36^ This provides the rationale for us to test US I‐PTT for neuroblastoma.

**SCHEME 1:**
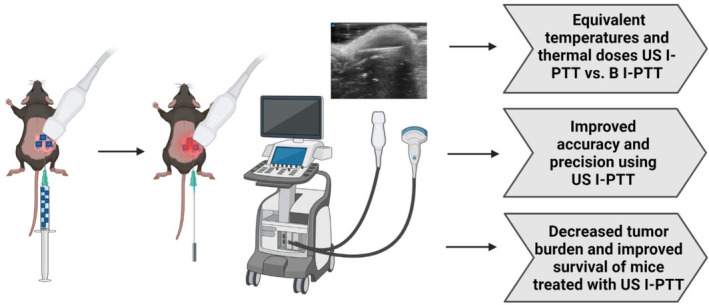
Ultrasound‐guided interstitial photothermal therapy (US I‐PTT) improves treatment outcomes in a 9464D neuroblastoma model. (Left–right) Workflow and outcomes for US I‐PTT. US I‐PTT is delivered by first injecting Prussian blue nanoparticles (PBNPs) (100 μL of 1 mg/mL) using a syringe needle fitted with a catheter under US image guidance for accurate placement in the center of the tumor of 9464D tumor‐bearing mice. The needle is then removed, and an optical laser fiber terminating in a cylindrical diffuser is inserted through the catheter using the ultrasound probe for accurate placement. Next, US I‐PTT is administered by illuminating the optical laser fiber to heat the target treatment area containing injected PBNPs. US I‐PTT generates equivalent temperatures and thermal doses as blinded, non‐image guided I‐PTT (B I‐PTT) but yields improved accuracy and precision of treatment, which results in decreased tumor burden and improved mouse survival compared to B I‐PTT.

In this study, we investigated the effect of US I‐PTT in comparison to blinded, non‐image‐guided I‐PTT (B I‐PTT) in the TH‐MYCN 9464D^37^ syngeneic murine model of neuroblastoma (referred to as 9464D hereafter). We hypothesized that US I‐PTT improves the accuracy of the placement of the laser fiber within a tumor and facilitates delivery of PTT with increased precision, yielding improved tumor treatment outcomes in terms of tumor regression and survival. Specifically, we treated C57BL/6 mice bearing 9464D tumors with US I‐PTT, B I‐PTT, or left them untreated and compared the in vivo photothermal heating, accuracy of treatment administration, tumor regression, tumor‐free and long‐term survival (Scheme 1). Through these studies, we seek to answer if US image guidance improves the in vivo efficacy of I‐PTT over B I‐PTT in the 9464D model of neuroblastoma, which would provide key feasibility data on the benefit of using US I‐PTT for improved tumor treatment outcomes.

## 2 RESULTS

### 2.1 US I‐PTT generates equivalent thermal doses as B I‐PTT in the 9464D neuroblastoma model

**FIGURE 1:**
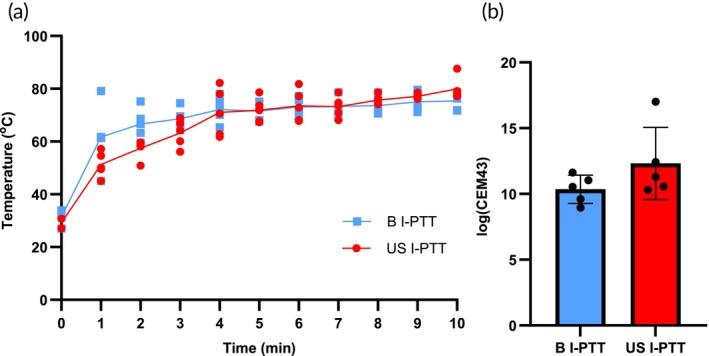
Ultrasound‐guided interstitial photothermal therapy (US I‐PTT) and blinded I‐PTT (B I‐PTT) generate equivalent temperatures and thermal doses in 9464D tumor‐bearing mice. (a) Average treatment temperature was measured externally using a FLIR thermal camera at 1‐min intervals for the B I‐PTT and US I‐PTT groups. (b) The average thermal doses (log(CEM43)) achieved during B I‐PTT and US I‐PTT in vivo (*p* = 0.175).

To examine whether US I‐PTT generated different photothermal heating profiles than B I‐PTT, we applied the treatments to 9464D tumor‐bearing mice and temporally measured the resulting temperatures for the 10‐min duration of treatment. Tumors of mice treated with B I‐PTT or US I‐PTT reached average final temperatures of 74.7 ± 2.9 and 80.0 ± 4.3°C, respectively, after 10 min of laser administration (Figures 1a and S2). Each 1‐min interval measurement was calculated to be statistically equivalent between the groups, using a standard *t*‐test. These heating curves were used to calculate the thermal dose administered to each mouse using the cumulative equivalent minutes at 43°C (CEM43) equation defined by Sapareto and Dewey.^38^ B I‐PTT and US I‐PTT generated statistically equivalent thermal doses in the 9464D tumors as measured by CEM43 (Figures 1b and S2). B I‐PTT generated an average thermal dose of 10.4 ± 1.1 log (CEM43), and US I‐PTT generated an average thermal dose of 12.3 ± 2.7 log (CEM43), which were not statistically different (*p*‐value = 0.175). These data suggest that US I‐PTT generated equivalent temperature profiles and thermal doses as compared to B I‐PTT.

### 2.2 US I‐PTT improves the accuracy of treatment delivery over B I‐PTT

**FIGURE 2:**
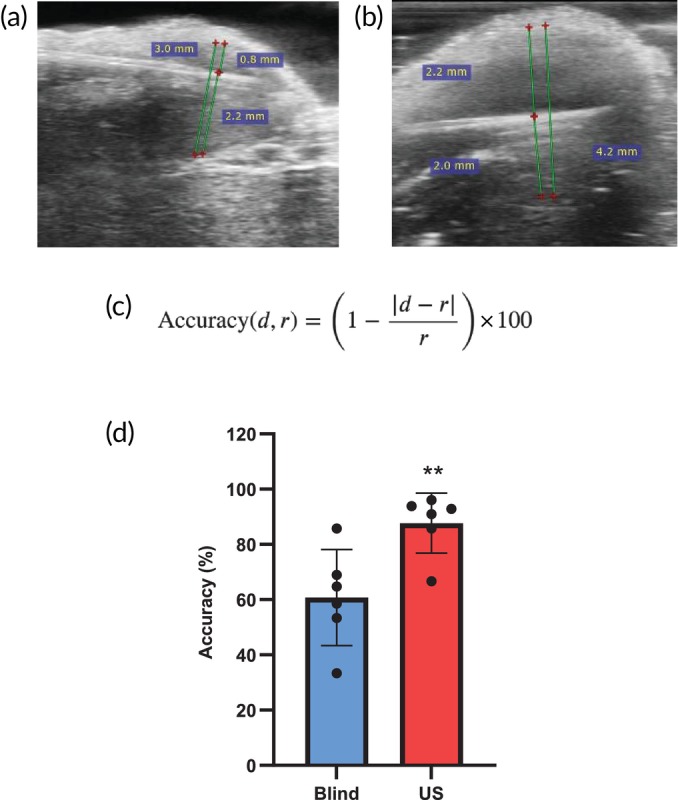
Ultrasound image guidance improves accuracy of interstitial photothermal therapy (I‐PTT) administration. (a, b) Annotated ultrasound images representative of the (a) blinded I‐PTT (B I‐PTT) and (b) ultrasound‐guided I‐PTT (US I‐PTT) groups. (c) Treatment accuracy was evaluated using the given equation; *r*: radius of the tumor, *d*: distance of the needle from the upper or bottom edge of the tumor. (d) The equation in (c) was used to calculate percent accuracy of treatment in the B I‐PTT and US I‐PTT groups; **p < 0.01.

Having established the photothermal heating capability of US I‐PTT, we sought to evaluate if US image guidance improves the accuracy of treatment administration over B I‐PTT. As such, we acquired US images of the initial needle placement (used to deliver both the PBNPs and the laser diffuser) for mice treated with both US I‐PTT (Figure 2a) or B I‐PTT (Figure 2b). Because US imaging was not used in the treatment scheme for B I‐PTT, these images were captured immediately after the needle was placed, strictly for accuracy measurements. These images were annotated using a DICOM file software (RadiAnt) (Figure S3).

To quantify the results visually attained by the US, we derived an equation to evaluate the accuracy of treatment (Figure 2c). In the equation, “*r*” represents the radius of the tumor and “*d*” represents the distance from the top or bottom edge of the tumor to where the needle was placed. The theoretical maximum for needle placement (100% accuracy) was defined as the radius value, as that represents the midpoint and most central location within the tumor. While we are limited in our description of accuracy to 2D image analysis of 3D tumors, this is a useful way to quantify our images and estimate the accuracy of treatment administration within the visualized planes obtained by US imaging. We determined that US I‐PTT was delivered significantly more accurately (87.7 ± 10.9%) than B I‐PTT (60.8 ± 17.4%), and with much higher reproducibility, as measured by the standard deviation of accuracy (10.8% vs. 17.4% for US I‐PTT vs. B I‐PTT). (Figure 2d). The significant increase in accuracy in the US I‐PTT group compared to the B I‐PTT group is a direct result of the US imaging allowing the PBNPs and laser fiber to be placed centrally within the tumor, as all other metrics were kept consistent between the treatment groups.

### 2.3 US I‐PTT triggers improved tumor regression, tumor‐free survival, and long‐term survival in the 9464D neuroblastoma model

**FIGURE 3:**
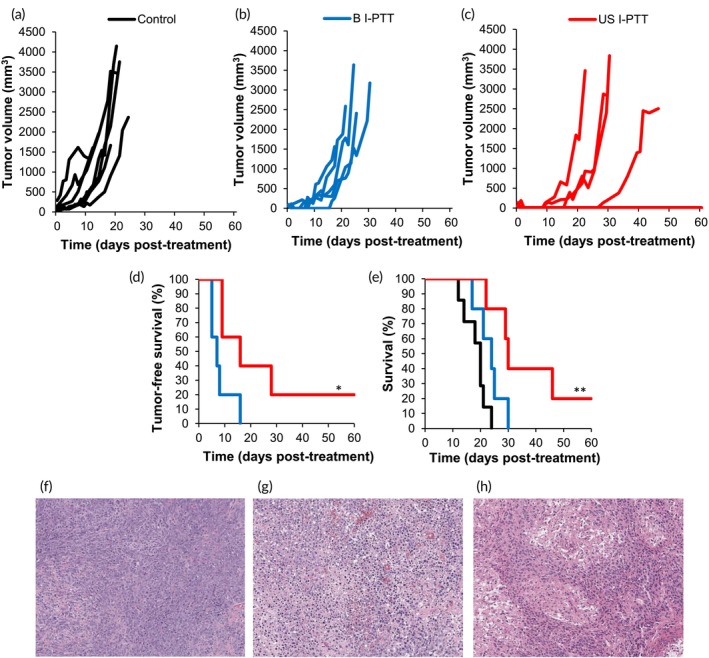
Ultrasound‐guided interstitial photothermal therapy (USI‐PTT) decreases tumor growth and improves mouse survival over blinded I‐PTT (B I‐PTT) and Control. (a–c) Individual tumor growth curves of mice (a) left untreated (Control), or treated with (b) B I‐PTT or (c) US I‐PTT. (d) Tumor‐free survival curves of mice treated with B I‐PTT (blue) or US I‐PTT (red). log‐rank test; *p < 0.05. (e) Overall survival curves of mice treated with B I‐PTT (blue) or US I‐PTT (red). **p < 0.01, compared to Control. (f–h) Hematoxylin and eosin‐stained tumor samples from mice (f) left untreated (Control), or treated with (g) B I‐PTT, or (h) US I‐PTT.

To determine the effect of image‐guided I‐PTT on survival and tumor regression in the 9464D neuroblastoma mouse model, we treated 9464D tumor‐bearing mice with US I‐PTT or B I‐PTT or left them untreated. Tumor growth was temporally monitored (Figure 3a–c), and the mice were humanely euthanized once tumors exceeded the endpoint tumor dimensions. Mice in the US I‐PTT group displayed slower growth kinetics (Figure 3c) than mice treated with B I‐PTT (Figure 3b) or left untreated (“Control”) (Figure 3a). Importantly, mice treated with US I‐PTT exhibited significantly (*p* = 0.031) improved tumor‐free survival (TFS) (median TFS: 16 days) compared to mice treated with B I‐PTT (median TFS: 7 days) (Figure 3d). Overall survival was also significantly improved in mice treated with US I‐PTT (median survival [MS]: 30 days; [*p* = 0.0035]) as compared to untreated mice (MS: 20 days) and marginally, though not statistically significantly better than B I‐PTT (MS: 24 days). By Day 40 post‐treatment, there was 40% overall survival in the US I‐PTT group as compared to 0% in both the B I‐PTT and control groups (Figure 3e). These survival results clearly suggest the critical role that the accurate delivery of I‐PTT has on treatment outcomes, as the improved accuracy of US I‐PTT resulted in improved median and overall survival and decreased tumor burden compared to the other groups.

Building on these results, we sought to investigate the histological effects of the treatments on the tumors. Using an additional cohort of mice distinct from the survival study described above, we euthanized mice at 4 or 8 h post‐treatment and collected the tumors for hematoxylin and eosin (H&E) analysis (Figures 3f–h and S4). Histological analysis revealed that tumors treated with US I‐PTT exhibited higher overall levels of necrosis and had greater infiltration of immune cells (e.g., neutrophils and macrophages) compared to the other groups. Based on the International Neuroblastoma Pathology Classification (INPC) scoring, the tumors scored equivalently across treatment groups and timepoints with all tumors being scored as poorly differentiated (PD) or undifferentiated (UD) (Table S1). However, the overall mitosis‐karyorrhexis index (MKI) scoring was higher in the tumors treated with B I‐PTT, with 80% of the tumors (*n* = 4/5) exhibiting high MKI (>4%). The control group also exhibited a high MKI in 100% of the tumors analyzed. Conversely, only 50% of tumors (*n* = 3/6) from the US I‐PTT group exhibited a high MKI. Additionally, the tumors in the US I‐PTT group had smaller nucleoli and less chromatin and cytoplasm present, indicative of a less aggressive tumor phenotype post‐treatment. We attribute these main differences in histology to the increase in accuracy provided by imaging in the US I‐PTT treatment scheme. We did not observe differences in the tumors harvested after 4 versus 8 h across treatment groups. Taken together, these findings demonstrate that US I‐PTT yielded more effective treatment outcomes in the 9464D neuroblastoma model.

## 3 DISCUSSION

In this study, we illustrated the feasibility and benefit of ultrasound guidance in improving treatment outcomes in a murine model of neuroblastoma. This study builds upon prior work that illustrated the benefit of I‐PTT over externally applied PTT (surface PTT; S‐PTT) to treat murine neuroblastoma. Previously, we observed that I‐PTT heated tumors more uniformly than S‐PTT, and this manifested in improved treatment outcomes of neuroblastoma tumor‐bearing mice.^3^ Despite these promising results, these studies were performed in subcutaneous models of murine neuroblastoma. In this context, the tumor is accessible from the outside for the injection of PBNPs and the interstitial laser fiber. Unfortunately, most neuroblastoma tumors arise deep within the abdomen in or around the adrenal glands and are therefore superficially inaccessible, rendering S‐PTT or I‐PTT impossible without image guidance. To address this limitation, we proposed the use of image guidance in conjunction with I‐PTT to facilitate the accurate delivery of the components of I‐PTT (i.e., PBNPs, laser fiber) within a deep‐seated neuroblastoma tumor.

As a first step to investigating this proposed technology, we presented the use of ultrasound image guidance in combination with I‐PTT to visualize a murine neuroblastoma tumor for accurate needle and laser fiber placement. As proof‐of‐concept, these studies were also performed in a subcutaneous model in order to obtain feasibility data for this treatment modality, namely US I‐PTT. Ongoing studies are examining the US I‐PTT platform in orthotopic neuroblastoma tumors in mice in vivo, which will more closely resemble its clinical presentation.

Ultrasound imaging is a favorable imaging modality, as it is a safe, non‐invasive, and inexpensive method for visualizing human anatomy.^39^ Ultrasound imaging is already commonly used in the clinical workup and treatment of various forms of cancer,^40^, ^41^ and would therefore be seamlessly integrated into the treatment scheme.^42^ Thus, we chose ultrasound imaging for the studies presented in this report. Despite the benefits of ultrasound imaging to I‐PTT that this paper demonstrates, there are still several clinical limitations we aim to address in future studies. Firstly, although ultrasound imaging extends the application of I‐PTT to deeper tumors, not all neuroblastoma tumors will be easily accessible and imageable by ultrasound and would thus not benefit from this approach. Additionally, ultrasound imaging provides a two‐dimensional visualization of the tumor, unlike other imaging modalities (e.g., magnetic resonance imaging) that provide an image in three dimensions and thus may not provide complete accuracy for needle and laser fiber placement. The in vivo application of US I‐PTT presented in these studies used an external thermal camera to measure heating kinetics. Admittedly, these temperature measurements may be measuring the surface of the animal and not accurately describe the heat inside the tumor. Because of this, we plan to incorporate an intratumoral thermocouple in future studies to accurately track tumor heating at multiple points within the tumor. Lastly, human neuroblastoma tumors will likely be irregular in shape and size, and may require more than one laser fiber to provide full treatment coverage. We anticipate that a personalized and mathematically simulated treatment planning scheme will take place prior to the application of the therapy, wherein multiple laser fibers will be placed throughout the tumor to ensure adequate and uniform heating as previously demonstrated in the literature for photodynamic therapy.^43^, ^44^ These preclinical studies are ongoing in the laboratory.

Overall in this study, we observed that US I‐PTT improved treatment outcomes (i.e., delayed tumor growth and improved survival) over B I‐PTT in mice bearing 9464D tumors (Figure 3). Because the two I‐PTT techniques achieved equivalent thermal doses (Figure 1), we attribute the improved outcomes of US I‐PTT to the increased accuracy of needle and laser fiber placement within the tumor, resulting in more precise PTT administration (Figure 2). Placing the laser more centrally may have resulted in a more uniform and complete heating within the tumor, thus delaying tumor growth. Overall, the findings of this study lay the foundation for future studies investigating the efficacy of US I‐PTT in treating deep‐seated, orthotopically inoculated 9464D tumors in vivo. We envision that US I‐PTT will be an effective treatment strategy for these deeper neuroblastoma tumors, as a first step toward clinical translation of US I‐PTT for patients with neuroblastoma and potentially other types of solid tumors.

## 4 MATERIALS AND METHODS

### 4.1 Cells

The transgenic murine neuroblastoma cell line TH‐MYCN 9464D^37^ was provided by Dr. Carol Thiele (Pediatric Oncology Branch, NIH, Bethesda, MD, USA). 9464D cells were cultured in Dulbecco's Modified Eagle's Medium (DMEM; ThermoFisher Scientific, Waltham, MA, USA) containing 10% fetal bovine serum (R&D Systems, Minneapolis, MN, USA), 1% nonessential amino acids (ThermoFisher Scientific), 0.5% antibiotic‐antimycotic (ThermoFisher Scientific), and 0.05% β‐mercaptoethanol (ThermoFisher Scientific).

### 4.2 PBNP synthesis

PBNPs were synthesized using a scheme as described previously.^2^, ^10^, ^19^ Briefly, citric acid (MilliporeSigma, Burlington, MA, USA) was added to both aqueous solutions of iron (III) chloride hexahydrate (FeCl_3_ · 6H_2_O; MilliporeSigma) (10 mM; 20 mL) and potassium hexacyanoferrate (II) (K_4_[Fe(CN)_6_] · 3H_2_O; MilliporeSigma) (10 mM; 20 mL), separately, at 400–600 rpm at 60°C to a final concentration of 5 × 10^−3^ m in each solution (MilliporeSigma). Once dissolved, the K_4_[Fe(CN)_6_] · 3H_2_O solution was added to the Fe(Cl)_3_ · 6H_2_O solution dropwise under stirring at 400–600 rpm. After stirring for 5 min at room temperature, the resulting particles were isolated by centrifugation in equal volumes of acetone (MilliporeSigma) and water (10,000 × *g* for 15 min at room temperature) and then rinsed/resuspended by microtip sonication (40% amplitude for 30 s) in Milli‐Q water (MilliporeSigma) using a Q500 sonicator (QSonica LLC, Newton, CT, USA). Isolation and rinse steps were repeated three times before the particles were finally resuspended in Milli‐Q water with sonication at the desired concentration. Two additional rinse steps were performed in ethanol prior to use in vivo. PBNPs were analyzed for concentration by oven‐drying particles at 80°C and measuring absorbance at 680 nm using a SpectraMax microplate reader (Molecular Devices, San Jose, CA, USA). The size distributions and surface charge of the PBNPs were characterized by dynamic light scattering and electrophoretic light scattering, respectively, on a Zetasizer Nano ZS (Malvern Instruments, Malvern, UK). The absorbance spectra of PBNPs were measured by Vis–NIR spectrophotometry on the Genesys 10S UV–Vis machine (ThermoFisher Scientific). PBNPs exhibited an average size (hydrodynamic diameter) of 295.3 nm, a zeta potential of −65.3 mV, and a characteristic absorbance peak at ~710 nm (Figure S1).

### 4.3 Ultrasound

The Visual Sonics Vevo 3100 high‐frequency small animal ultrasound (FUJIFILM VisualSonics, Toronto, Canada) with the MX550S transducer was used for these studies. All imaging was performed in B‐mode.

### 4.4 Animals

All animal studies were conducted in accordance with protocols approved by the Institutional Animal Care and Use Committee (IACUC) of the George Washington University (protocol # A2023‐83) and in accordance with the humane care of research animals. For tumor growth and survival studies, 5‐week‐old female C57BL/6 mice were purchased from Jackson Laboratory (Bar Harbor, ME) and acclimated for a week prior to handling. Female mice were used in this study for consistency because 9464D cells were established in female mice,^37^, ^45^ although we acknowledge the importance of considering sex as a biological variable.

### 4.5 Mouse neuroblastoma model

Syngeneic TH‐MYCN neuroblastoma mouse models were established by suspending 1 million 9464D cells in 100 μL of 1:1 PBS/high concentration Matrigel (Corning, Tewksbury, MA, USA) and subcutaneously injecting into the shaved backs of 6–7 weeks old female C57BL/6 mice. Tumors were treated once volumes reached a size of 5 mm (44–60 mm^3^).

### 4.6 Interstitial laser fiber setup

To set up the I‐PTT system, a SMA‐905 fiber coupler (Laserglow Technologies, Toronto, Canada) was mounted onto a NIR collimated diode laser system (808 nm; Laserglow Technologies), and an optical fiber was then attached to the fiber coupler. The optical fiber was fitted with a 5 mm cylindrical terminal diffuser (LifePhotonic, Bonn, Germany; numerical aperture: 0.22, core diameter: 200 μm). The output power was measured using the IS6‐D‐Vis divergent beam detector (#7Z02488, Ophir Optronics, North Logan, UT, USA) connected to the Juno RHS meter (#7Z01250, Ophir Optronics) and visualized using the StarLab v3.62 software.

### 4.7 In vivo study design

These methods have been adapted from our previous work.^3^ When the 9464D tumors reached a dimension of 5 mm in length or width (44–60 mm^3^ in volume), the animals were randomly distributed into three treatment groups: (1) Control (receiving no treatment), (2) B I‐PTT (receiving i.t. injection of 100 μL of 1 mg/mL PBNPs and irradiated with interstitial NIR laser for 10 min without ultrasound image guidance), and (3) US I‐PTT (receiving i.t. injection of 100 μL of 1 mg/mL PBNPs and irradiated with the optical fiber with a 5 mm terminal diffuser coupled to the NIR laser for 10 min with ultrasound image guidance). Randomization was performed using the “minimization” strategy, using initial tumor volume as a continuous nuisance variable to balance the influence of tumor burden on outcome.^46^, ^47^ Mice were anesthetized prior to treatment using 2%–5% isoflurane. Specific methods for US I‐PTT are described below. After treatment, mouse health, tumor sizes, and tumor‐free and overall survival were monitored and recorded daily. Mice were humanely euthanized according to the approved IACUC protocol through CO_2_ narcosis followed by cervical dislocation when tumor diameter measured 20 mm in one dimension or no later than a week after tumor ulcerations were first observed.

### 4.8 In vivo US I‐PTT

For US I‐PTT, the Vevo 3100 imaging system was used to visualize tumors and placement of the needle (for PBNP delivery) and the laser fiber. Specifically, a U‐100 29G 1 mL/cc 1/2″ (12.7 mm; BH Supplies, Berwick, PA, USA) needle was inserted through a shielded IV catheter (inner diameter of 0.98 mm and outer diameter of 1.3 mm, BD Biosciences), and ultrasound imaging was used to ensure the needle entered the core of the tumor as described above. Then, PBNPs (100 μL of 1 mg/mL) were injected i.t. After injection, the needle was removed, and the catheter was secured and stabilized using medical tape for delivery of the laser fiber at the same location. The optical fiber, with a 5 mm terminal cylindrical diffuser (which was coupled to the NIR laser as described above), was then inserted through the catheter so that the terminal diffuser was positioned within the tumor. Once the fiber was properly placed, the laser was turned on and the laser power was adjusted accordingly to attain a temperature~75°C for 10 min. Temperature was monitored with a thermal camera (FLIR E5‐XT, Arlington, VA, USA), and laser power was adjusted over the treatment period to maintain a superficial temperature range, as specified. Thermal doses administered were calculated and represented as the logarithm of the CEM at 43°C (log(CEM43)) over 10 min, with the CEM43 calculated as previously described.^38^ For B I‐PTT, PBNPs (100 μL of 1 mg/mL) were i.t. injected, and the laser fiber was placed through the catheter in a similar manner, but in the absence of ultrasound image guidance.

### 4.9 Histological analysis

A separate cohort of 12 C57BL/6 mice was used for histological analysis 4 or 8 h post‐treatment to observe morphological differences present between two different timepoints and each treatment group. Once tumors reached a dimension of 5 mm in length or width, mice were randomly divided into five groups (*n* = 2‐3/group): (1) Control (untreated); (2) B I‐PTT 4 h (euthanized 4 h post‐B I‐PTT); (3) US I‐PTT 4 h (euthanized 4 h post‐US I‐PTT); (4) B I‐PTT 8 h (euthanized 8 h post‐B I‐PTT); or (5) US I‐PTT 8 h (euthanized 8 h post‐US I‐PTT). Once the mice were euthanized, their tumors were excised, fixed in zinc‐buffered formalin for 24 h, and sent to HistoWiz (Brooklyn, NY, USA) for sectioning and staining. A board‐certified pathologist scored one slide cut in equivalent positions within the tumor for MKI, INPC, and gross histology characteristics (i.e., coagulative/fat necrosis or presence of Homer‐Wright rosettes) as a means of characterizing their overall pathology (Table S1). These analyses were done in collaboration with Dr. Julie Feldstein from HistoWiz.

### 4.10 Statistics

Results obtained in this study are expressed as mean ± standard deviation. Statistical significance between groups (i.e., thermal dose and accuracy) was evaluated with an unpaired *t*‐test, taking *p* values <0.05 as significant. TFS and overall survival were analyzed according to Kaplan–Meier curves and log‐rank tests. Statistical significance throughout the study is indicated as n.s.: non‐significant, *p < 0.05, **p < 0.01. Statistical tests were performed using the Prism GraphPad 10.0.1 software.

## 5 CONCLUSIONS

This study represents a meaningful advance toward clinical translation of I‐PTT for the treatment of neuroblastoma. We demonstrate that US I‐PTT generated statistically equivalent thermal doses as B I‐PTT in vivo. US I‐PTT facilitated more accurate treatment administration, which resulted in significantly decreased tumor burden, improved tumor‐free and long‐term survival over mice treated with B I‐PTT. Ongoing studies are investigating the use of US I‐PTT in orthotopic models of murine neuroblastoma to model its use in a deep‐seated tumor setting.

## AUTHOR CONTRIBUTIONS

**Grace E. Olsson:** Conceptualization; data curation; methodology; investigation; validation; formal analysis; writing – original draft; writing – review and editing. **Rohan V. Patil:** Conceptualization; data curation; formal analysis; validation; methodology; writing – review and editing; investigation. **Samantha J. Chin:** Data curation; investigation; writing – review and editing. **Katharine N. Rus:** Data curation; investigation; writing – review and editing. **Elizabeth E. Sweeney:** Formal analysis; supervision; validation; writing – original draft; writing – review and editing. **Karun V. Sharma:** Conceptualization; methodology; investigation; supervision; writing – review and editing; project administration; formal analysis. **Rohan Fernandes:** Conceptualization; methodology; investigation; validation; formal analysis; supervision; funding acquisition; visualization; writing – original draft; writing – review and editing; resources; project administration.

## CONFLICT OF INTEREST STATEMENT

Elizabeth E. Sweeney and Rohan Fernandes are co‐founders of ImmunoBlue, a biotechnology company focused on developing PBNP‐based nanoimmunotherapies.

## Supporting information


**Data S1.** Supporting Information.

## Data Availability

The data that support the findings of this study are available from the corresponding author upon reasonable request.
