# Supplementary material for: Ultrasound‐guided interstitial photothermal therapy generates improved treatment responses in a 9464D model of neuroblastoma
Source: Bioeng Transl Med. 2025 Jan 6;10(2):e10749. doi: 10.1002/btm2.10749 (PMC11883107; doi:10.1002/btm2.10749)
Supplement: Supplementary file 1 — Data S1. Supporting Information. [file BTM2-10-e10749-s001.pdf]

SUPPORTING INFORMATION

Ultrasound-guided interstitial photothermal therapy generates improved treatment responses in a 9464D model of neuroblastoma

Grace E. Olsson[1], Rohan V. Patil[1,2], Samantha J. Chin[1,3], Katharine N. Rus[1], Elizabeth E. Sweeney[1,4], Karun V. Sharma[1,5], Rohan Fernandes[1,3,6]

[1] George Washington Cancer Center, School of Medicine and Health Sciences, George Washington University, Washington, DC, USA

[2] School of Medicine and Health Sciences, George Washington University, Washington, DC, USA

[3] The Integrated Biomedical Sciences Program, School of Medicine and Health Sciences, George Washington University, Washington, DC, USA

[4] Department of Biochemistry & Molecular Medicine, School of Medicine and Health Sciences, George Washington University, Washington, DC, USA

[5] Department of Interventional Radiology, Children's National Hospital, Washington, DC, USA

[6] Department of Medicine, School of Medicine and Health Sciences, George Washington University, Washington, DC, USA

* Correspondence to: 800 22nd St. NW, Washington, DC 20052; rfernandes@gwu.edu

This document contains **Figures S1-S4** and **Table S1**

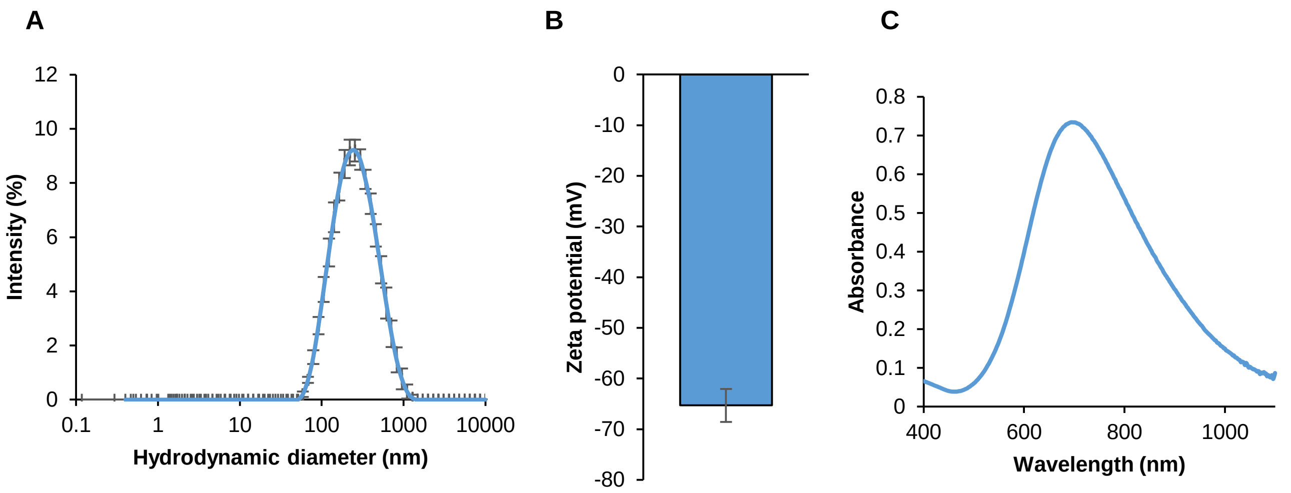

Supporting Information Figure S1. PBNP characterization. A) Size distributions was measured by dynamic light scattering. B) Surface charge (zeta potential) was measured by electrophoretic light scattering. C) Absorbance spectrum was measured by Vis-NIR spectrophotometry.

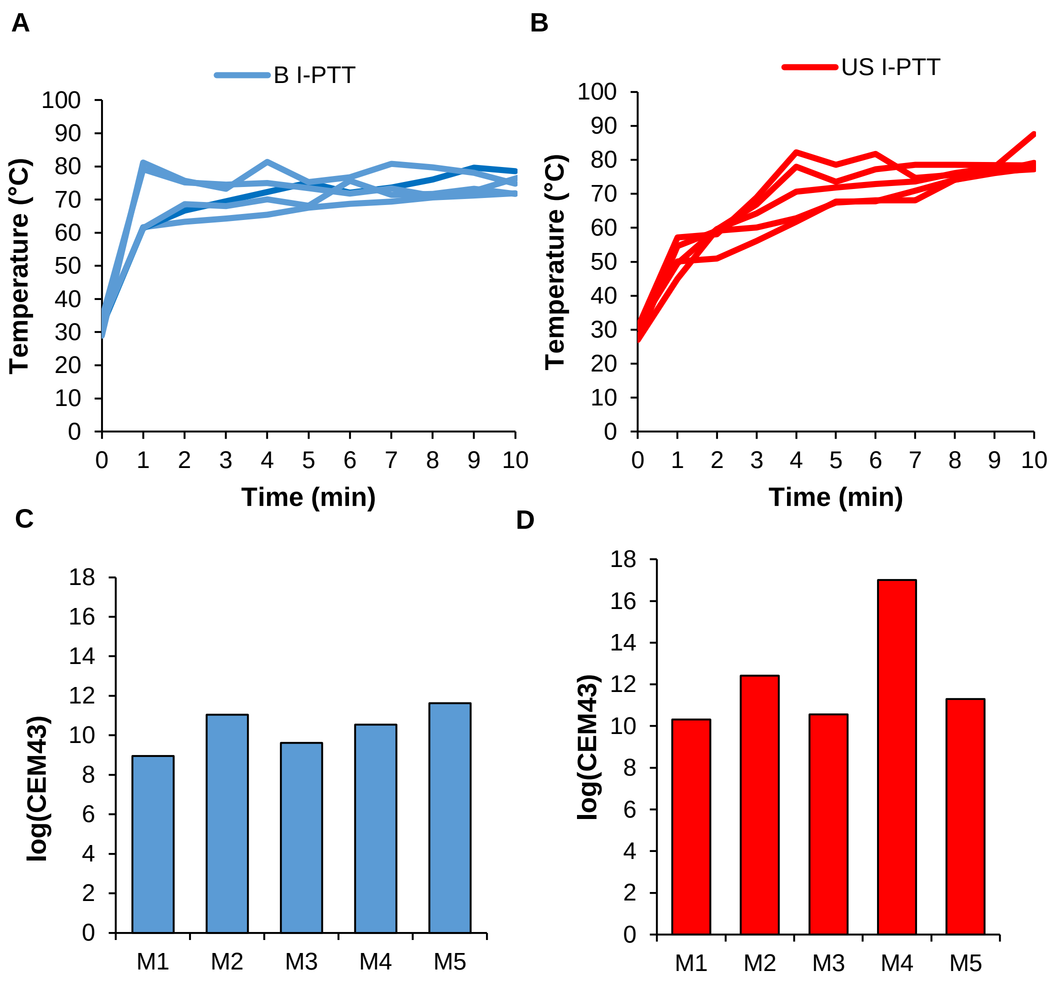

Supporting Information Figure S2. Heating curves and thermal doses for individual mice treated with B I-PTT or US I-PTT. A-B) Average treatment temperature was measured externally using a FLIR thermal at one-minute intervals for mice (n=5/group) treated with (A) B I-PTT or (B) US I-PTT groups. Each line represents one mouse. C-D) Thermal doses (log(CEM43)) achieved during (C) B I-PTT or (D) US I-PTT *in vivo.* M1-M5 indicate individual mice in each treatment group.

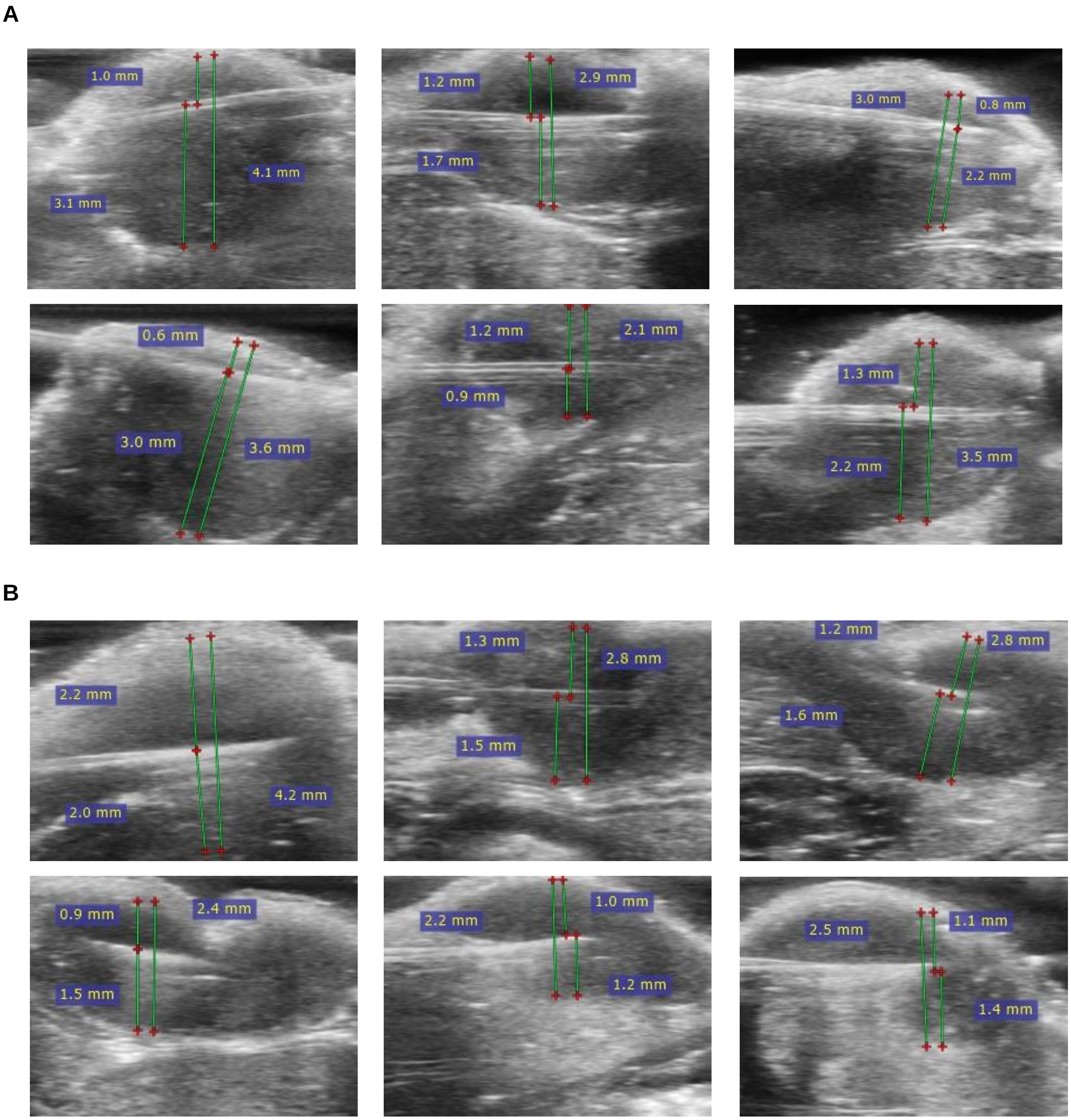

Supporting Information Figure S3. Tumor ultrasound images used for calculating the accuracy of B I-PTT and US I-PTT. Annotated ultrasound images for individual mice (n=6/group) treated with (A) B I-PTT or (B) US I-PTT.

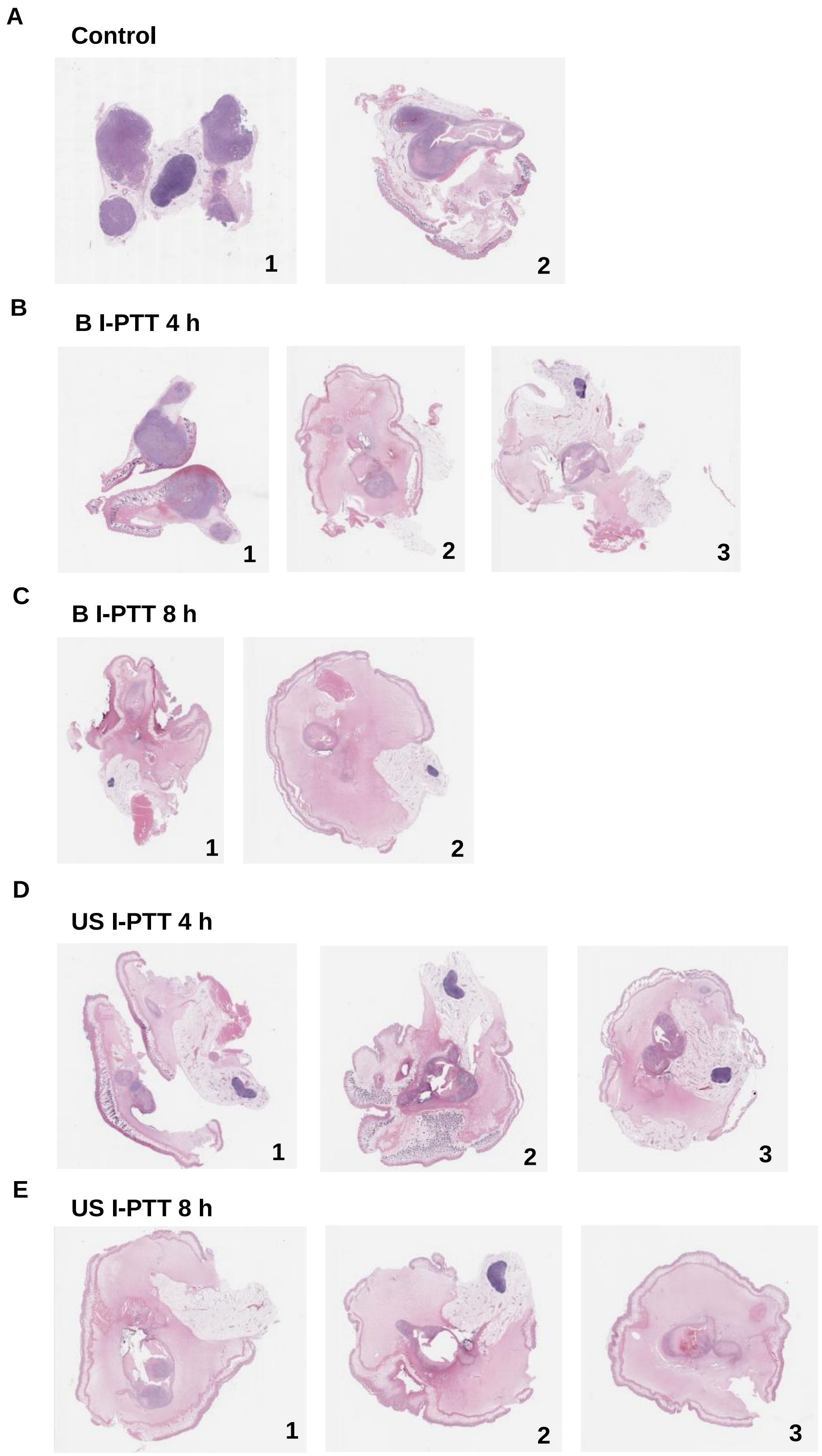

Supporting Information Figure S4. Histological analysis of harvested 9464D tumors post-treatment. Tumors from mice (A) left untreated (Control) or treated with B I-PTT and harvested after (B) 4 h or (C) 8 h, or treated with US I-PTT and harvested after (D) 4 h or (E) 8 h were stained with H&E and evaluated by a pathologist.

Supplementary Information Table S1. Histopathology analysis of harvested tumors

Sample	MKI	INPC	Necrosis coagulative/fat
Control (1)	>4% high	PD, stroma rich	1
Control (2)	>4% high	PD	1
B I-PTT 4 h (1)	>4% high	PD	1
B I-PTT 4 h (2)	>4% high	PD	2
B I-PTT 4 h (3)	>4% high	UD	2
B I-PTT 8 h (1)	<2% low scanty tumor	PD	2
B I-PTT 8 h (2)	>4% high	UD	2
US I-PTT 4 h (1)	>4%	PD, UD	2
US I-PTT 4 h (2)	2-4% intermediate	UD	3
US I-PTT 4 h (3)	<2% low	PD	3
US I-PTT 8 h (1)	>4% high	UD	2
US I-PTT 8 h (2)	2-4% intermediate	PD	3
US I-PTT 8 h (3)	>4% high	UD	2

MKI: mitosis-karyorrhexis index

INPC: International Neuroblastoma Pathology Classification scoring

PD: poorly differentiated; UD: undifferentiated
